# Early Surgery Prolongs Professional Activity in IDH Mutant Low-Grade Glioma Patients: A Policy Change Analysis

**DOI:** 10.3389/fonc.2022.851803

**Published:** 2022-03-09

**Authors:** Pierre A. Robe, Matea Rados, Wim G. Spliet, Reinier G. Hoff, Peter Gosselaar, Marike L. D. Broekman, Martine J. van Zandvoort, Tatjana Seute, Tom J. Snijders

**Affiliations:** ^1^ University Medical Center (UMC) Utrecht Brain Center, Department of Neurology and Neurosurgery, University Medical Center Utrecht, Utrecht, Netherlands; ^2^ Departement of Clinical Neuropsychology, University of Utrecht, Utrecht, Netherlands; ^3^ Department of Pathology, University Medical Center Utrecht, Utrecht, Netherlands; ^4^ Department of Anesthesiology, University Medical Center Utrecht, Utrecht, Netherlands

**Keywords:** low-grade glioma, early resection, professional activity, awake craniotomy, return to work

## Abstract

**Background:**

Until 2015, Dutch guidelines recommended follow-up and biopsy rather than surgery as initial care for suspected low-grade gliomas (LGG). Given evidence that surgery could extend patient survival, our center stopped following this guideline on January 1, 2010 and opted for early maximal safe resection of LGG. The effects of early surgery on the ability of patients to work remains little documented.

**Methods:**

A total of 104 patients operated on at our center between January 2000 and April 2013 and diagnosed with the WHO 2016 grade 2 astrocytoma, IDH mutant or oligodendroglioma, IDH mutant and deleted 1p19q were included. The clinical characteristics, survival, and work history of patients operated on before or after January 2010 were obtained from the patients’ records and compared. The minimal follow-up was 8 years.

**Results:**

As per policy change, the interval between radiological diagnosis and first surgery decreased significantly after 2010. Likewise, before 2010, 25.8% of tumors were initially biopsied, 51.6% were resected under anesthesia, and 22.5% under awake conditions versus 14.3%, 23.8%, and 61.9% after this date (*p* < 0.001). The severity of permanent postoperative neurological deficits decreased after 2010. In total, 82.5% of the patients returned to work postoperatively before 2010 versus 100% after 2010. The postoperative control of epilepsy increased significantly after 2010 (74.4% vs. 47.9%). The median time from diagnosis to a definitive incapacity to work increased by more than 2 years after 2010 (88.7 vs. 62.2 months).

**Conclusion:**

A policy shift towards early aggressive surgical treatment of IDH mutant LGG is safe and prolongs the patients’ ability to work.

## Introduction

WHO grade 2 low-grade gliomas (LGG) often affect professionally active young adults (1). They grow slowly and seldom provoke debilitating symptoms initially. However, as these tumors progress, they induce significant disabilities and death, with a median survival of 139 months for oligodendrogliomas and 67 months for astrocytomas (2–4).

The adjuvant treatment, follow-up, and prognosis of gliomas depend on their histological type and molecular characteristics. Despite progress in metabolic imaging, radiologic techniques can neither reliably differentiate low- from high-grade gliomas nor determine their complete molecular profiles (5–7). Historically, stereotactic biopsies were reported to yield inappropriate or inconclusive histological results in up to 38% of cases, due to tumor heterogeneity, and still carry a significant risk of transient morbidity (2%–9.6%) and permanent morbidity and mortality (0.4%–0.9%) (8–11). While major genetic (IDH1/2 mutations, 1p19q codeletions) and epigenetic [e.g., MGMT promoter methylation (12)] changes seem more homogenously distributed in tumors than histologic alterations, it remains unclear whether this is the case for more specific but important changes like CDKN2A/B homozygous deletions or EGFR amplification (13) and how often sampling errors can occur with (serial) biopsies. Upfront debulking surgery does not only allow for a broader tumor sampling but also results in cytoreduction, which has been arguably associated with a survival advantage as compared with upfront biopsy (14–18). As a result, debulking is increasingly recommended rather than stereotactic biopsy in guidelines for the upfront diagnostic and decision making in these tumors (19, 20).

Despite these recommendations, the extent of the surgical management of suspected LGG remains a matter of active debate (21–23). Despite advances in intraoperative mapping techniques that have considerably reduced morbidity of resections (18, 24–26), their long-term morbidity has been little studied. Patients indeed generally show a decline in cognitive function and quality of life following surgery for gliomas (27, 28). As a result, early/larger resections could prematurely alter patients’ neurologic/cognitive function and quality of life in the period prior to disease progression, as compared with biopsies.

Until April 2015, The Dutch guidelines recommended the follow-up of suspected LGGs without alarming symptoms and to simply biopsy patients with neurologic symptoms other than seizures or atypical radiologic findings (e.g., contrast enhancement). Debulking surgery was advised only in patients with radiologic progression, mass effect, or intractable seizures (16, 29–32).

Based on the abovementioned potential survival and sampling quality arguments, our center opted on January 1, 2010 to deviate from the then implemented Dutch national guidelines and to rather advocate the early maximal safe resection of suspected LGG, as recommended by the North American NCCN guideline (19). Accordingly, operable patients were proposed a maximal safe resection within 3–6 months of their first consultation at our center, even in the case of asymptomatic or seemingly stable disease. Biopsies were reserved to cases unamenable to a debulking due to a deep-seated location or patient refusal of more extensive surgery. This complete and timely defined change of policy allows to compare the short- and long-term benefits or drawbacks of primarily following-up and biopsying versus primarily being surgically more aggressive in molecularly defined low-grade gliomas.

We report here—with a minimal follow-up of 8 years for all patients—the effects of our change of policy on patient survival, return to work, and duration of professional activity following diagnosis.

## Methods

### Study Population

This study was approved by the medical ethical committee of the University Medical Center of Utrecht (as part of protocol # 16-342). The need for informed consent was waived by the ethics committee of the UMC Utrecht for this retrospective analysis of data and material collected as part of routine clinical care. Using our institutional pathology database, we retrospectively identified all adult patients (≥18 years, *n* = 205) operated on at our center for a supratentorial LGG between January 1, 2000 and April 30, 2013. We revised the pathological slides, charts, and preoperative imaging and attempted to classify the tumors molecularly according to the recent WHO classifications of tumors. To this end, the available slides and pathology reports (histology and molecular biology) were thoroughly reviewed, and for older tumors lacking molecular testing, we could obtain formalin-fixed, paraffin-embedded (FFPE) cores from 101 tumors from our institution’s pathology department. These were processed in tissue microarrays and stained for IDH1-R132C, ATRX, H3K27M, and H3K27me3 according to Filipski et al. (33). Twelve additional FFPE samples were further processed for next-generation sequencing (NGS) and copy number variation (CNV) analysis by our pathology department laboratory, using their standard operating procedures, and yielding conclusive results in 10 cases. As this item was not a standard at the time of this study design and revision of the pathology slides, the CDK2N/B status of most tumors was not ascertained, and for this reason, tumors were classified according to the WHO 2016 and not the WHO 2021 taxonomy.

A total of 101 tumors were excluded after these steps: 14 patients who had been operated on prior to 2000, 21 patients who had actually been diagnosed prior to 2000, 25 with radiological gliomatosis cerebri as defined on MRI (i.e., extending diffusely to 3 or more lobes), one DNET, 6 glioblastoma, IDH wild type, one PMXA grade 2, one grade 1 diffuse glioma NEC, one H3K27mutant (high grade) diffuse pediatric glioma, and 21 A2 NOS, OA2 NOS, and O2 NOS tumors.

An additional 10 patients who were known at our center prior to July 2009 but were merely followed up and only operated on after 2010 were also excluded from the main analyses in order to avoid cross-over bias between our cohorts of patients.

Surgical procedures were defined as biopsy, tumor resection, and tumor resection under awake conditions. Two patients who underwent resection within 3 months after biopsy were included in the “resection” and “awake resection” groups, respectively.

### Outcomes

Surgical morbidity was defined as any adverse event that occurred within 30 days of surgery and classified according to the Common Terminology Criteria for Adverse Events (CTCAE) v3.0 (24).

Tumor volumes were segmented measured from the pre- and postoperative MRI images using the Brainlab Origin planning server software. Volumetric diagnostic and preoperative MRI were available in 88/104 and 97/104 patients, respectively. Tumor volumes were considered unchanged following biopsies, and postoperative MRI volumes were assessed on imaging performed within 3 months of the surgery. Tumors were classified topographically according to Sawaya for their eloquence (34).

Survival data were obtained from the patient charts and verified on May 21, 2021 providing a follow-up of at least 8 years for the entire patient population. Overall survival (OS) was measured as the time between the first diagnostic imaging showing the tumor and death or censoring. Survival data were censored at last follow-up for patients still alive at that moment. Two patients were lost to follow-up at some time after their surgery and were censored at that time.

The work history—a standard part of the follow-up of patients at our center—was retrieved from the medical and social documents of the patients’ charts. The time to loss of productivity was measured from the date of the first imaging diagnosis until the date when the patient had completely stopped to work or died. Patients who retired for nonmedical reasons while still active were censored on their date of retirement.

### Adaptation of the Surgical Policy

**Figure 1:**
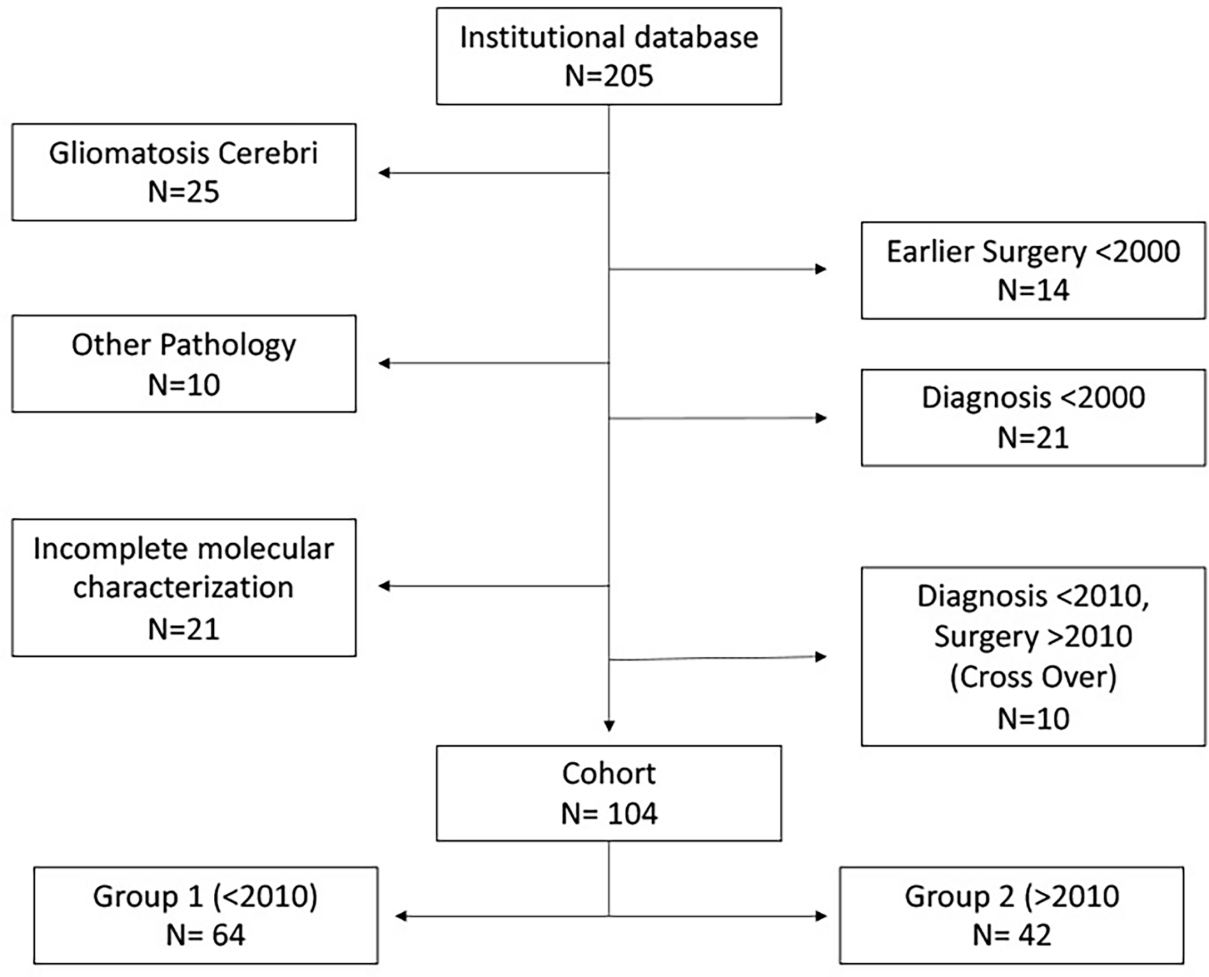
Flow chart of the patient selection for groups 1 (before 2010) and 2 (after 2010).

Our center switched acutely on January 1, 2010 from the Dutch national guidelines that then recommended the follow-up of suspected LGG until growth or progression and biopsy rather than surgery for diagnostic confirmation (13) towards the early and maximal safe resection of these lesions. As a result, patients were primarily proposed resection within 3–6 months of their first consultation even in the case of asymptomatic or stable diseases. The indication for surgical resection was set by the treating neurosurgeon in all patients with suspected LGG, unless the surgeon expected that no meaningful extent of resection could be obtained and/or patient refused resection. This change of policy allowed us to define two cohorts of patients: group 1 (operated on prior to January 1, 2010) and group 2 (after this date). Ten patients who were known at our center prior to July 2009 but were merely followed up and only operated on after 2010 were excluded from the main analyses, in order to avoid cross-over bias (**Figure 1**).

The surgical procedures are described in detail in the **Supplementary Methods**.

### Analyses and Statistics

Categorical data were analyzed using Pearson’s Chi-square (*χ* ^2^) test or Fisher’s exact test based on sample size. Nonparametric continuous data were analyzed using Mann–Whitney *U* tests. Parametric continuous data were analyzed using an independent-sample *t*-test.

Differences in survival and the time to definitive stop of work (for the patients that were professionally active at the time of diagnosis) between groups were first quantified in a univariable analysis with the log-rank test. Multivariable analyses were performed using Cox’s proportional hazards regressions.

Analyses were performed using SPSS_v25_ (IBM, Chicago, IL, USA) and Prism_v9_ (GraphPad, San Diego, CA, USA) with two-sided statistical significance defined at *p* < 0.05.

## Results

### Study Population

The study population included 104 adult patients with a histologically confirmed supratentorial WHO grade 2 astrocytoma (IDH1/2 mutant, ATRX mutant, *n* = 70) or oligodendroglioma (IDH1/2 mutant, 1p19q codeleted, *n* = 34). Clinical and demographic characteristics are summarized in **Table 1**.

**Table 1:** Clinical and demographic characteristics of the patients at the time of radiological diagnosis.

	Total	Group 1 (January 1, 2000–December 31, 2009)	Group 2 (>January 1, 2010)	*p*-value
**Patients [*n* (%)]**	104	62 (59.6%)	42 (40.4%)	
**Gender (M/F)**	64/40	35/27	29/13	0.195 (*χ* ^2^)
**Age at diagnosis [mean (range)]**	42 (21–72)	43 (23–72)	40 (21–69)	0.282 (*t*-test)
**KPS at time of diagnosis (range)**	90 (60–100)	90 (60–100)	90 (60–100)	0.350 (*χ* ^2^)
**Presentation**				
**Epileptic seizures**	86.5%	88.7%	83.3%	0.431 (*χ* ^2^)
**Incidental finding**	6.7%	6.5%	7.1%	0.890 (*χ* ^2^)
**Location**				
**Left hemisphere**	50 (48.1%)	26 (41.9%)	24 (57.1%)	
**Both hemispheres**	8 (7.7%)	5 (8.1%)	3 (7.1%)	0.303 (*χ* ^2^)
**Right hemisphere**	46 (44.2%)	31 (50%)	15 (35.7%)	
**Eloquence (Sawaya)**				
**Class I (noneloquent)**	19 (18.3%)	14 (22.6%)	5 (11.9%)	
**Class II (near eloquent)**	37 (35.6%)	21 (33.9%)	16 (38.1%)	0.384 (*χ* ^2^)
**Class III (eloquent)**	48 (46.1%)	27 (43.5%)	21 (50%)	
**Volume (in cm^3^, mean and range)**	56.9 (3.5–244.5)	50.8 (3.5–244.5)	63.5 (7.4–185.6)	0.221 (*t-*test)
**Pathology**				
**Astrocytoma, IDH mutant**	70 (67.3%)	43 (69.4%)	27 (64.3%)	0.589 (*χ* ^2^)
**Oligodendroglioma, IDH mutant and 1p19q codeleted**	34 (32.7%)	19 (30.6%)	15 (35.7%)	
**Worked at time of diagnosis**				
**Yes**		51 (82.3%)	39 (92.8%)	
**No**		9 (14.5%)	2 (4.8%)	0.267 (*χ* ^2^)
**Unknown**		2 (3.2%)	1 (2.4%)	

A biopsy was performed in 22 patients (21.2%), while 82 (88.8%) patients underwent primary resection. An awake resection was performed in 40 of these patients (48.8%).

Prior to imaging diagnosis, 90/104 (86.5%) of patients had been professionally active, 8 had retired at that time, 1 had stopped for unrelated medical reasons, and 2 had never been professionally active (missing data: *n* = 3% or 2.9%). These proportions were similar in both groups (*p* = 0.23, *χ* ^2^ test). A description of the jobs performed by the patients is provided in **Supplementary Table S1**.

### Change of Policy

There was no significant difference in the age at diagnosis, symptoms at presentation, epilepsy at presentation, histology, location or eloquence [according to Sawaya (34)] and volume at diagnosis of the tumors, or the frequency of direct adjuvant chemotherapy or radiotherapy between patients from group 1 (prior to 2010, *n* = 62) and group 2 (after 2010, *n* = 42). The KPS at time of diagnosis was also similar in both groups (range: 60–100, *p* = 0.350, *χ* ^2^ test, **Tables 1**, **2**).

**Table 2:** Initial treatment in the entire cohort of patients and in both groups 1 and 2.

	Total	<January 1, 2010	>January 1, 2010	*p*-value
**First surgical procedure**				
**Awake resection**	40 (38.5%)	14 (22.6%)	26 (61.9%)	
**Asleep resection**	42 (40.4%)	32 (51.6%)	10 (23.8%)	<0.001 (*χ* ^2^)
**Biopsy**	22 (21.1%)	16 (25.8%)	6 (14.3%)	
**Direct adjuvant therapy**				
**None**	79 (76%)	49 (79%)	30 (71.4%)	
**Radiotherapy**	20 (19.2%)	11 (17.7%)	9 (21.4%)	0.496 (*χ* ^2^)
**Chemotherapy**	4 (3.8%)	1 (1.7%)	3 (7.1%)	
**Combined**	1 (0.9%)	1 (1.7%)	0 (0%)	

Eighty-eight percent of the patients were operated on within 6 months of their first diagnostic imaging after 2010 versus only 61.3% prior to this date (*p* = 0.0028, *χ* ^2^ test) and the median follow-up time prior to surgery decreased from 140 to 76 days after 2010 (log rank: *p* = 0.001; **Supplementary Figure S1**). In the period between the diagnostic imaging and the first surgery, the tumor volumes increased by a mean of 32.6% (SD: 77.7%; range: 0%–395%) prior to 2010 versus only 9.8% (SD: 42.4%; range: 0%–279%) after this date (*p* = 0.012, Mann–Whitney *U* test). The KPS of the patients at the time of their first surgery had not worsened significantly in any of the groups as compared with their KPS at time of diagnosis (*p* = 0.857 and >0.9999, respectively, Wilcoxon test).

Before January 1, 2010, 25.8% of patients primarily underwent biopsy versus only 14.3% after this date, while the proportion of awake craniotomies increased from 22.6% before 2010 to 61.9% later (*p* < 0.001, *χ* ^2^ test). The extent of resection prior and after 2010 for the debulking operations did not differ significantly (79.6% vs. 74.3%, *p* *=* 0.262, *t*-test), and the residual postoperative tumor volumes after debulking operations likewise did not differ between both groups (14.3 ± 15.1 cm^3^ vs 14.6 ± 16.2 cm^3^, *p* = 0.945, *t*-test).

### Surgical Morbidity

There was no difference in the general surgical morbidity between patients from groups 1 and 2 (**Table 3**). There were one postoperative hemorrhage and one postoperative arrhythmia in group 2, and two postoperative surgical infections in group 1. No patient developed pulmonary embolism or deep venous thrombosis within 3 months from surgery, and there was no mortality in this time period.

**Table 3:** Postoperative morbidity of the patients operated before and after January 1, 2010.

	Before 2010	After 2010	*p*-value
**No complication**	**44 (71%)**	**30 (71.4%)**	0.959 (*χ* ^2^ test)
**General complications**			
**Bleeding**	0	1	
**Cardiac arrhythmia**	0	1	0.135 (*χ* ^2^ test)
**Wound infection**	2	0	
**Temporary neurological deficits**			
**Dysphasia**	7	3	0.515 (Fisher’s test)
**Motor**	1	2	
**Permanent neurological deficits**			
**Dysphasia**	7	1	
**Motor**	1	1	**0.0275** (*χ* ^2^ test)
**Visual field defect**	0	3	

Neurological deficits were considered permanent when still present 3 months after the surgery.

The incidence of transient and permanent neurological deficits did likewise not differ significantly [permanent deficits: 8 (12.9%) in group 1 and 5 (11.9%) in group 2 (NS, *χ* ^2^ test)]. However, the nature and severity of the permanent neurological deficits were significantly different before and after 2010. They consisted of dysphasia (11.3% vs 2.9%), paresis (1.6% vs 2.4%), and visual field defects (0% vs. 7.1%; *p* *=* 0.0275, *χ* ^2^ test for the whole). The CTCAE_v3.0_ grade of these deficits was significantly milder after 2010 (*p* =0.046, *χ* ^2^ test, **Table 4**). The visual field defects in particular were of grade 1 (asymptomatic) in two patients and 2 (symptomatic, not interfering with activities of daily living) in one.

**Table 4:** Severity of the postoperative neurological deficits according to the Common Terminology Criteria of Adverse Events (CTCAE v3.0).

CTCAE v3.0	Before 2010 (group 1)			After 2010 (group 2)		
	Grade 1	Grade 2	Grade 3	Grade 1	Grade 2	Grade 3
**Permanent dysphasia**		3	4		1	
**Permanent motor**		1			1	
**Visual field defect**				2	1	

The numbers represent the number of patients suffering each given type of deficit.

### Survival

The median overall survival estimate from the time of diagnosis was 152.6 months (95% CI [123.8–181.5]) for the entire cohort and differed significantly between IDH mutant astrocytomas and oligodendrogliomas, IDH mutant and 1p19q deleted (respectively 115.2 and 176.1 months, *p* *=* 0.002, log-rank test). It was significantly different between patients who had been merely biopsied and those who underwent a craniotomy (under general anesthesia or awake), with respective survival medians of 123.9 and 159.8 months (*p* =0.047, log-rank test). In Cox multivariable analysis, the histology, tumor volume at diagnostic, and the type of surgery (biopsy vs. debulking) significantly influenced the overall survival in our cohort, in contrast to the age and KPS at the time of diagnostic (**Supplementary Table S2**).

**Figure 2:**
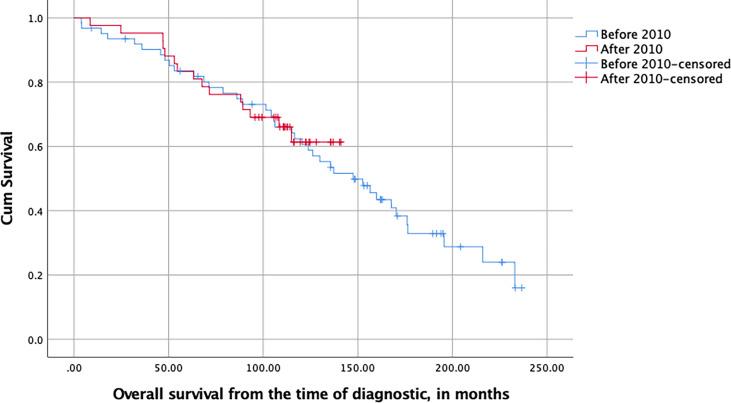
Kaplan–Meier plot of the overall survival of LGG patients stratified with respect to their date of surgery and measured in months from the time of first diagnostic imaging (NS, log-rank test).

With respect to our change of policy, the overall survival (as measured from the time of diagnosis) of patients diagnosed before or after 2010 was similar (*p* = 0.808, log-rank test, **Figure 2**).

### Return to Work, Duration of Ability to Work, and Epilepsy Outcome

Of the 51 group 1 patients who had been professionally active prior to their diagnostic, 10 (19.6%) quit working following their diagnostic but prior to their first surgery versus only 1 out of 39 in group 2 (2.56%, *p* *=* 0.014, *χ* ^2^ test). After their first surgery, of those patients who still worked, 33/41 (80.48%) returned to work after their operation in group 1 while 100% returned to work after their surgery in group 2. This difference in the rate of return to work was significant (*p* = 0.004, *χ* ^2^ test). As mentioned, the employment status of two patients of group 1 and one patient of group 2 prior to their diagnosis was missing. We thus performed a sensitivity analysis with all missing values of group 1 being reallocated as patients who would have worked preoperatively and returned to work postoperatively and the missing values of group 2 as not having returned to work. Even in this exaggerated scenario, the difference between both groups remained significant with respect to the rate of return to work (81.4% vs. 97.4%, *p* = 0.0203, *χ* ^2^ test).

**Figure 3:**
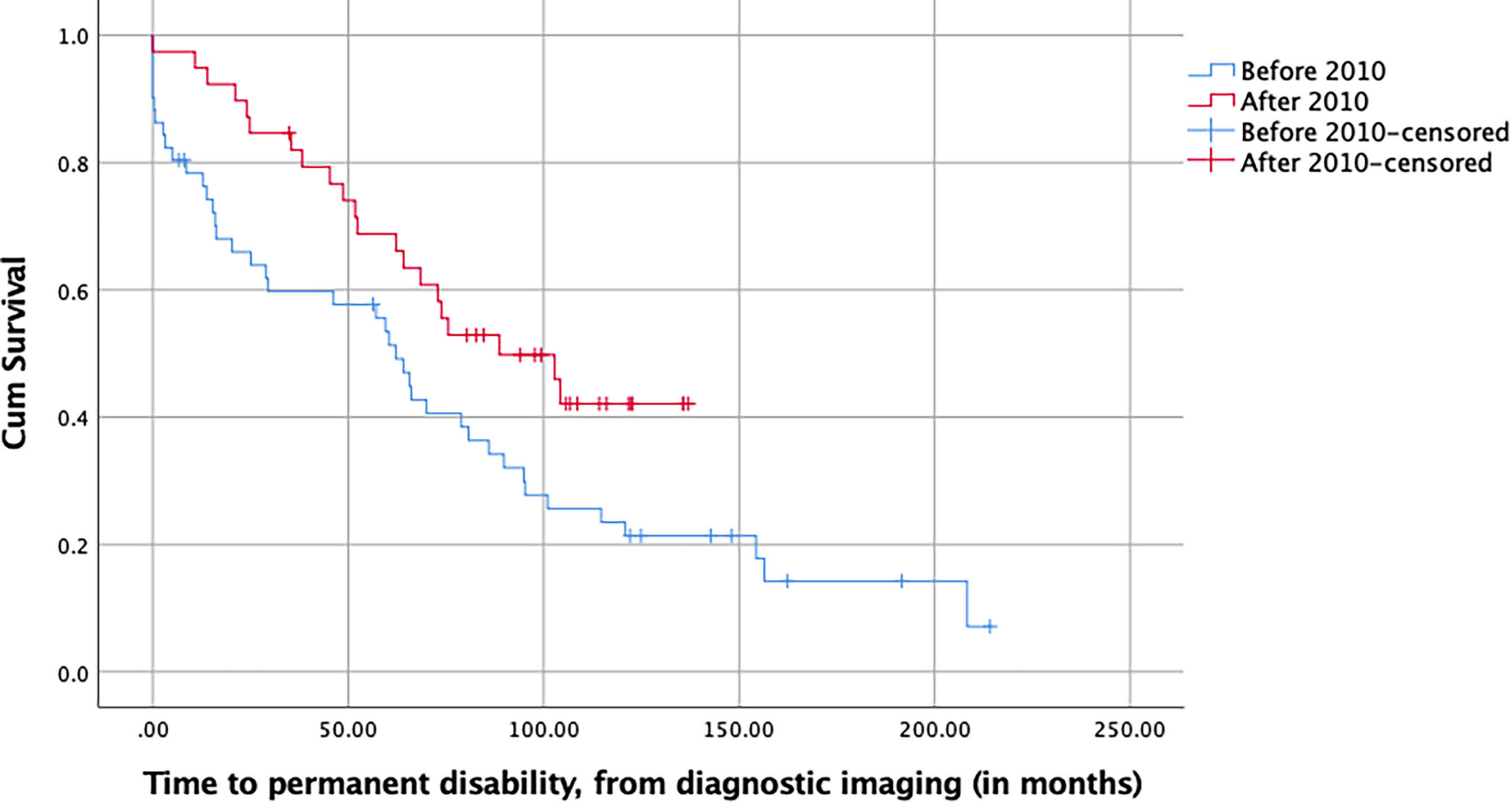
Kaplan–Meier plot of the time to permanent work disability of LGG patients stratified with respect to their date of surgery and measured in months from the time of first diagnostic imaging (*p* = 0.030, log-rank test).

Of the patients who were active at the time of diagnosis, and counting from that moment on, those of group 1 permanently lost their ability to work significantly sooner than those of group 2, with a median time to permanent work disability of 62.2 months for group 1 and 88.7 months for group 2 (*p* = 0.030, log-rank test, **Figure 3**). This difference remained significant in a multivariable Cox model taking the gender, age, KPS, history of seizures, and tumor volume at diagnosis, as well as the type of surgery, histology, and postoperative treatment into account (*p* = 0.027, HR = 0.451, 95% CI [0.223–0.911], **Table 5**).

**Table 5:** Multivariable (Cox) analysis of the time to the permanent loss of ability of patients to work (measured form the time of diagnostic imaging).

Variables in the equation	Sig.	HR	95% CI for Exp(B)	
			Lower	Upper
**Pre-post-2010**	**0.027**	0.451	0.223	0.911
**Gender (male vs. female)**	0.262	0.678	0.344	1.337
**Age at diagnostic imaging**	0.474	1.013	0.977	1.051
**Epileptic seizures at diagnostic (no vs. yes)**	0.067	2.322	0.941	5.727
**KPS at diagnostic**	**0.038**	0.958	0.921	0.998
**Volume (in cm^3^) at diagnostic**	**0.006**	1.01	1.003	1.016
**Pathology (astrocytoma vs. oligodendroglioma)**	0.063	2.083	0.96	4.521
**Early postop treatment: XRT**	0.989			
**Early postop treatment: none**	0.951	1.031	0.392	2.71
**Early postop treatment: PCV**	0.596	0.536	0.053	5.375
**Early postop treatment: Stupp**	0.965	0.95	0.099	9.158
**Early postop treatment: TMZ**	0.833	0.834	0.154	4.527
**Type of surgery: debulking**	0.036			
**Type of surgery: biopsy**	0.244	1.534	0.747	3.15
**Type of surgery: awake craniotomy**	**0.01**	4.008	1.396	11.507

Significant values are highlighted in bold.

Of note, at the time of censoring for work disability, 25/48 patients in group 1 (52.1%, unknown status for 3 patients) suffered from clinically active epilepsy (i.e., other than Engel class I) versus 10/39 in group 2 (25.6%, *p* = 0.012, *χ* ^2^ test). This difference remained significant when—as a sensibility analysis—the 3 patients with unknown status in group 1 were all assumed to be free from epilepsy (*p* = 0.0301, *χ* ^2^ test).

## Discussion

A growing body of literature suggests a survival advantage of the early and aggressive surgical treatment of LGGs, including Evidence-Based Medicine level 2 data (14, 35). LGGs however tend to develop in young, professionally active adults. The question remains whether the aggressive surgery, while increasing survival, could result in additional morbidity and in an earlier loss of economic productivity in those young patients.

Our well-defined, thorough, and dated change of policy between a conservative treatment protocol (based on a follow-up and advocating biopsies rather than debulking) towards an early maximal safe resection of LGGs allowed us to retrospectively answer this question. Several potential biases were avoided. First, in order to avoid cross-contamination of our two cohorts (cross-over bias), we included only those patients diagnosed between specific periods of time corresponding to the two policies. Patients diagnosed during the first period (“conservative” treatment) but operated on during the second (“aggressive” treatment) period, were thus discarded from the analyses. Second, the change of policy was not associated with any shift in the demographics (at the time of diagnostic of the disease) of patients, in the allocation to adjuvant therapies, or in the nature of these adjuvant therapies. Third, our population of patients consists purely of fully characterized IDH mutant grade 2 gliomas according to the WHO 2016 classification of tumors (oligodendroglioma, IDH mutant and 1p19q codeleted or astrocytomas, IDH mutant), thus avoiding contamination bias by IDH wild-type or other types of grade 2 gliomas. This *post-hoc* selection of patients based on molecular profile differs from previous studies on surgical strategy for “presumed LGGs” (radiological diagnoses), in which beneficial results of aggressive surgery were driven—in part—by (molecularly) higher-grade tumors; this study, rather, is limited to true IDH-mutated LGGs.

Altogether, this observational study effectively constitutes a “split-wedge” design, in which the results reflect the effects of a more aggressive surgical approach on the professional functioning of LGG patients with minimal bias.

The switch of policy was effective, as demonstrated by the significantly reduced delay between diagnosis and surgery on the one hand and the 1.8-fold reduced percentage of biopsies since January 1, 2010, with a 2.75-fold increase in awake debulking. Interestingly however, only 25.8% of the patients underwent a biopsy prior to 2010. This can be inherent to the delay between diagnosis and first surgery in this group. Indeed, patients suffered significant increases in tumor volumes between the diagnosis and surgery in this group, with potentially more mass effect, a criterion for debulking in the then valid treatment guidelines.

The switch towards a policy of early maximal safe resection of LGG has thus consisted of the earlier operation of patients, more debulking in place of biopsies and more awake craniotomies. It did not result in more radical resections, as both the extent of resection and residual volumes postdebulking (excluding the biopsies) remained similar after 2010. This change of policy did not at all alter the overall survival of our patients, as measured from the time of imaging diagnosis. This can be due to the relatively low proportion of biopsies in our first cohort (14), as well as the similar residual tumor volumes postdebulking in both cohorts (35). It could also in part be due maybe to the absence of “hidden malignant tumors” in our molecularly defined cohorts of patients, as compared maybe with these previous reports on the effect of radical resection, but in agreement with other recent observations (36). Altogether, the observed survival of our patients in both cohorts was in line with the literature (37).

Survival with preserved quality of life is of utmost importance in LGG patients (21). The abundant use of awake craniotomies since January 2010 helped us reduce the severity of *de novo* permanent neurological postoperative morbidity. Altogether, the severity of permanent neurological complications decreased after 2010 and, at a maximal severity level of 2, never limited the activities of patients. These results agree with published data on the safety of tumor resections performed under neuromonitoring (24) and further support the value of maximal safe surgical strategies against LGG.

Patients treated since 2010 were also significantly more likely to return to work postoperatively than those diagnosed before, with respectively 100% and 80.48% of patients returning to their professional lives postoperatively. These results are in line with the literature. In a recent series of 25 patients with glioma operated on under awake conditions and neuromonitoring, Mandonnet et al. indeed found that 80% of patients could return to work postoperatively (38), while in a prospective, more recent prospective cohort of 74 patients, Ng and collaborators described a rate of 97.1% of return to work following surgery for low-grade gliomas (39). In addition, a significant number of group 1 patients quit working in the period between their diagnosis and their surgery versus only one in group 2 (19.6% vs. 2.6%).

In the course of their disease, patients of group 2 also remained professionally active significantly longer than patients of group 1, with a hazard ratio of 0.429, i.e., a risk to losing one’s job divided by more than two, translating in an increase of more than 2 years of their median active survival. This increase in professionally active survival remained significantly different between both groups when controlling for other relevant parameters like gender, age, KPS, epilepsy at presentation, pathology, and early postoperative treatment. Another factor that could have played a role in the risk of becoming permanently disabled would be a time-dependent change in social rules, criteria, or legislation regarding the health-related fitness to work. Major changes in this respect did however not happen in the Netherlands during the period studied, as confirmed by the statistics of the National Office of Statistics, which do not report any significant increase in disease-related leaves of absence between 2008 and 2014 (40). A potential explanation for the longer ability to work for patients of group 2 could be that more aggressive surgeries reduced the physical or cognitive burden of their brain tumors. Indeed, at the time patients definitively stopped working (or were censored if still active at the last follow-up), group 2 patients were significantly more often completely seizure-free (Engel class I) than those operated on prior to this date (74.4% vs. 47.9%). Patients with a biopsy were also significantly more likely to present symptomatic seizures at this time than those who underwent a debulking (*p* *=* 0.001, *χ (* 2) test). These finding agrees with previous literature that showed a correlation between surgical aggressiveness and epilepsy control in low-grade gliomas (41), as well as with the inverse correlation between the duration of seizure history and postoperative seizure control (42).

The limitations of our study stem from the retrospective nature of our data collection. As a result, 25 A2 NOS, OA2 NOS, and O2 NOS histological grade 2 tumors were excluded from our analyses. Of these, 16 worked at the time of diagnosis. A sensitivity analysis of the professionally active survival of all patients, including those 16 patients, however confirms the very significant improvement that occurred after our policy change (median 88.7 months after 2010 vs. 59.7 months before, *p* *=* 0.006, log rank). In spite of the retrospective data collection, employment data were missing in only 2.9% of the patients, i.e., 2 patients in group 1 and 1 in group 2. This is unlikely to have altered our findings, as shown by our sensitivity analysis for the rate of return to work: even if those patients had all returned to work prior to 2010 and had become disabled when operated on after this date, the rate of postsurgical return to work still significantly increased after 2010. Another limitation is that allocation protocols to adjuvant treatments and tumor classifications have evolved significantly in the recent years. Our cohorts however precede the introduction of molecular data in the pathological armamentarium (our last patients were operated in April 2013), and the allocation of patients to adjuvant treatments, based on the then valid classification, has little changed between 2000 and 2013. To tackle this issue completely however, we extensively reviewed all tumors according to the WHO 2016 guidelines, selected out only fully characterized tumors for our analyses, and performed sensitivity analysis that incorporated the not otherwise specified—NOS—tumors. In addition, there was no difference between the distribution of postoperative adjuvant treatments between our two cohorts of patients.

Our study also has important strengths. First, it avoids selection bias by comparing two groups of patients defined by the thorough and dated introduction of a new surgical policy. Second, cross-over bias was also eliminated by this design and by discarding all the patients whose diagnostic and treatment did not both take place in the same defined period. Such a comparison of treatment strategies—rather than comparing groups according to treatment performed—reflects well the potential benefit of transitioning from delayed to early surgery in the clinical practice. Third, the long follow-up (minimum 8 years) of patients allows to draw matured conclusions pertinent to this slowly evolving disease.

In conclusion, a combination of early treatment and maximal use of awake craniotomies results in less serious postoperative deficits and lower epileptic burden in grade 2 astrocytomas, IDH mutant and oligodendrogliomas, IDH mutant and 1p19q codeleted as compared with a delayed, more conservative treatment strategy. As a corollary, patient remained able to work for a median of 2 years longer after their diagnosis following our change from a delayed conservative to an early “maximal safe” surgical strategy.

## Data Availability Statement

The original contributions presented in the study are included in the article/**Supplementary Material**. Further inquiries can be directed to the corresponding authors.

## Ethics Statement

This study was approved by the medical ethical committee of the University Medical center of Utrecht (as part of protocol # 16-342). The need for informed consent was waived by the Ethics Committee of the UMC Utrecht for this retrospective analysis of data and material collected as part of routine clinical care.

## Author Contributions

PR: conception, data accrual, analysis, writing, and funding. MR: data accrual and analysis. WS: data accrual and pathology review. RH: data accrual. PG: data accrual and review of the manuscript. MB: data accrual and review of the manuscript. Martine van Zandvoort: data accrual and review of the manuscript. TaS: data accrual, review of the manuscript, and funding. TJS: conception, data accrual, analysis, and review of the manuscript. All authors listed have made a substantial, direct, and intellectual contribution to the work and approved it for publication.

## Funding

This work was supported by an unrestricted grant of the T&P Bohnenn Fund for Neuro-Oncology Research to TaS and PR.

## Conflict of Interest

The authors declare that the research was conducted in the absence of any commercial or financial relationships that could be construed as a potential conflict of interest.

The handling editor declared a past coauthorship/collaboration with one of the authors (TS).

## Publisher’s Note

All claims expressed in this article are solely those of the authors and do not necessarily represent those of their affiliated organizations, or those of the publisher, the editors and the reviewers. Any product that may be evaluated in this article, or claim that may be made by its manufacturer, is not guaranteed or endorsed by the publisher.

## Supplementary Material

Supplementary Table 1 Methods - Surgical procedure.

Supplementary Figure 1 Kaplan-Meier plot of the duration of patient follow-up form the diagnosis until the first surgery.

## Abbreviations

LGG, low-grade glioma; DNET, dysembryoplastic neuroepithelial tumor; PMXA, pilomyxoid astrocytoma; IDH, isocitrate dehydrogenase; A2, astrocytoma, grade 2; OA2, oligoastrocytoma, grade 2; O2, oligodendroglioma, grade 2; NOS, not otherwise specified; NEC, not else classified.
