# Supplementary material for: Early Surgery Prolongs Professional Activity in IDH Mutant Low-Grade Glioma Patients: A Policy Change Analysis
Source: Front Oncol. 2022 Mar 9;12:851803. doi: 10.3389/fonc.2022.851803 (PMC8959843; doi:10.3389/fonc.2022.851803)
Supplement: Supplementary Table 1 — Methods - Surgical procedure. [file Table_1.docx]

| Type of Work | Before 2010 | After 2010 |
| --- | --- | --- |
| Accountant | 2 | 0 |
| Administrative employee | 2 | 0 |
| Administrative officer | 2 | 0 |
| Advisor in ergonomy | 0 | 1 |
| Bar employee | 0 | 1 |
| Car salesman | 1 | 1 |
| Car store owner | 0 | 1 |
| Carpenter | 1 | 0 |
| Cashier in a supermarket | 1 | 0 |
| CEO and owner of a midsize fabric | 0 | 1 |
| Chef (restaurant) | 1 | 0 |
| Chemistry teacher | 1 | 0 |
| Client manager (real estate) | 0 | 1 |
| Co-owner of a flower store | 1 | 0 |
| Coach for mentally deficient people | 1 | 0 |
| Coffee strore owner | 0 | 1 |
| Concrete specialist (buil | 1 | 0 |
| Construction employee | 0 | 1 |
| Consultant in automatisation | 1 | 0 |
| Consultant- self employed | 1 | 0 |
| Contract manager | 1 | 0 |
| Cook | 0 | 1 |
| Dental assistant | 0 | 1 |
| Dentist | 1 | 0 |
| District attorney | 1 | 1 |
| Employee | 3 | 2 |
| Employee in an IT company | 1 | 0 |
| Employee in a cinema | 0 | 1 |
| Employee in a flower plantation | 1 | 0 |
| Employee in a home for mentally deficient people | 0 | 1 |
| Employee in a horseriding school | 1 | 0 |
| Employee in a metal factory | 0 | 1 |
| Employee in an alimentary factory | 1 | 0 |
| Employee in the oil industry | 1 | 0 |
| Engineer | 0 | 1 |
| Export manager (commerce) | 0 | 1 |
| Freelance translator | 0 | 1 |
| Furniture maker (employee) | 0 | 1 |
| Highschool teacher | 1 | 0 |
| Hospital manager | 0 | 1 |
| House painter | 1 | 0 |
| Housecleaner | 1 | 0 |
| IC nurse | 1 | 0 |
| insurance company officer | 1 | 0 |
| Interieur designer | 1 | 0 |
| IRS employee | 1 | 0 |
| IT consultant | 1 | 0 |
| IT network manager | 1 | 0 |
| IT officer | 1 | 0 |
| IT specialist | 2 | 0 |
| Jurist | 0 | 1 |
| Management officer | 1 | 0 |
| Manager (technical company) | 1 | 0 |
| Manual worker | 0 | 1 |
| Manual worker in road construction | 0 | 1 |
| Marketing manager | 1 | 0 |
| Mason | 1 | 0 |
| MD (Family practitionner) | 0 | 2 |
| MD in the pharmaeutical industry | 1 | 0 |
| MD (specialist) | 0 | 1 |
| Mechanic | 0 | 1 |
| Middle manager in a supermarket | 1 | 0 |
| Military officer | 0 | 1 |
| Musician | 0 | 1 |
| Nurse in urology | 1 | 0 |
| Officer | 1 | 0 |
| Owner of a small company | 1 | 0 |
| Pediatric nurse | 1 | 0 |
| Pharmacy assistant | 1 | 0 |
| Photograph | 0 | 1 |
| Physical trainer (school) | 1 | 0 |
| Primary school treacher | 1 | 0 |
| Receptionist | 1 | 0 |
| Researcher in musical science | 1 | 0 |
| Restaurant employee | 0 | 1 |
| Restaurant owner | 0 | 1 |
| Roofer | 0 | 1 |
| Sailor | 1 | 0 |
| Sales officer | 1 | 0 |
| Sales representant | 1 | 0 |
| Salesforce in a supermarket | 0 | 1 |
| Salesman in a grocery store | 1 | 0 |
| Saleswoman in a flower sh | 0 | 1 |
| Saleswoman in a toy store | 1 | 0 |
| Self-employed social work | 1 | 0 |
| Social worker | 1 | 0 |
| Sport coach | 1 | 0 |
| Student worker | 0 | 1 |
| Technician  in the army | 1 | 0 |
| Technician in electronics | 1 | 0 |
| Travel agent | 1 | 0 |
| Truck driver | 1 | 1 |
| Vetereinary assistant | 0 | 1 |
| Work coach (employee) | 0 | 1 |
