# Supplementary figures and images for: Early Surgery Prolongs Professional Activity in IDH Mutant Low-Grade Glioma Patients: A Policy Change Analysis
Source: Front Oncol. 2022 Mar 9;12:851803. doi: 10.3389/fonc.2022.851803 (PMC8959843; doi:10.3389/fonc.2022.851803)

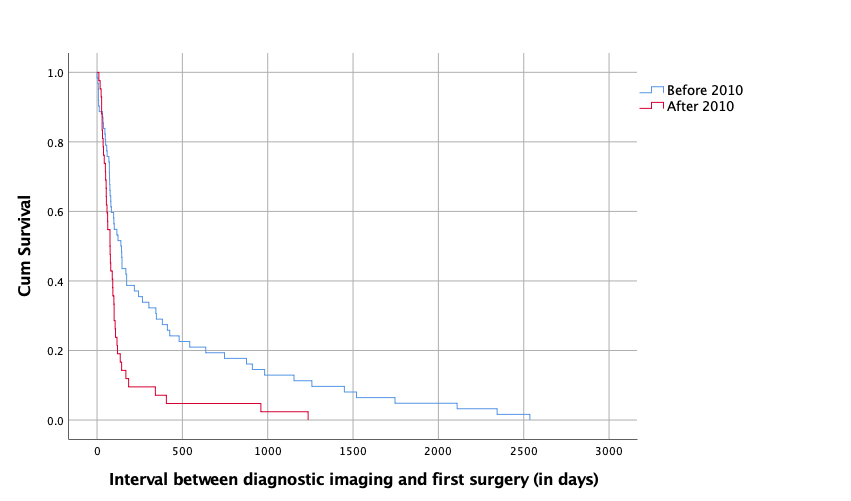

Supplement: Supplementary Figure 1 — Kaplan-Meier plot of the duration of patient follow-up form the diagnosis until the first surgery. [file Image_1.tif]
