# Supplementary material for: Early Surgery Prolongs Professional Activity in IDH Mutant Low-Grade Glioma Patients: A Policy Change Analysis
Source: Front Oncol. 2022 Mar 9;12:851803. doi: 10.3389/fonc.2022.851803 (PMC8959843; doi:10.3389/fonc.2022.851803)
Supplement: Supplementary file 3 [file Table_2.docx]

| **Variables in the Equation** | *P* value | **Hazard Ratio (HR)** | **95.0% CI for HR** | |
| --- | --- | --- | --- | --- |
|  |  |  | **Lower** | **Upper** |
| **Age** | 0.232 | 1.016 | 0.990 | 1.044 |
| **KPS** | 0.162 | 0.970 | 0.930 | 1.012 |
| **Histology (Astrocytoma vs. Oligodendroglioma)** | <0.001 | 6.710 | 2.657 | 16.950 |
| **Epileptic seizures at diagnostic (No vs. Yes)** | 0.160 | 1.933 | 0.771 | 4.846 |
| **Type First Surgery (Biopsy vs. Debulking)** | 0.021 | 2.545 | 1.149 | 5.635 |
| **Volume at presentation** | 0.047 | 1.008 | 1.000 | 1.015 |

**Table S2:** Cox multivariable model analysis of the overall patient survival (all patients considered)
